# Demographic History and Genetic Diversity of Rātā Moehau (*Metrosideros bartlettii*; Myrtaceae), a Critically Endangered Aotearoa New Zealand Endemic Tree

**DOI:** 10.1002/ece3.73352

**Published:** 2026-04-15

**Authors:** Jessie M. Prebble, Natalie J. Forsdick, Duckchul Park, Alexander J. F. Verry, Emma Simpkins, Thomas R. Buckley

**Affiliations:** ^1^ New Zealand Institute for Bioeconomy Science Manaaki Whenua – Landcare Research Group Lincoln New Zealand; ^2^ Genomics Aotearoa Dunedin New Zealand; ^3^ New Zealand Institute for Bioeconomy Science Manaaki Whenua – Landcare Research Group Auckland New Zealand; ^4^ Auckland Botanic Gardens Auckland New Zealand

**Keywords:** Aotearoa New Zealand, demographic modelling, genome assembly, Myrtaceae, Myrtle rust

## Abstract

Trees are a critical part of terrestrial ecosystems, but a large proportion of the world's trees are at risk of extinction. Targeted conservation efforts will be crucial to maintaining these species in the face of climate change and other anthropogenic impacts. When populations become small, they are increasingly susceptible to the negative genetic impacts of small population size and inbreeding. Understanding demographic history and genetic diversity of threatened species can facilitate the development of effective conservation management plans. Rātā Moehau (*Metrosideros bartlettii,* Myrtaceae) is an example of a Critically Endangered Aotearoa New Zealand endemic tree treasured by Māori, the Indigenous Peoples of Aotearoa. With just 14 wild individuals remaining, we implemented a study to assess the genetic diversity and infer demographic history to inform conservation management. We have sequenced and assembled the first genome for rātā Moehau, and generated resequencing data for wild and cultivated individuals. Using demographic reconstruction, we inferred a long‐term decline in population size over the past 1 million years. Further, cultivated individuals were found to have reduced diversity compared with wild individuals. Quantifying these genetic characteristics provides useful information for restoration decision‐making by conservation practitioners, led by local iwi (tribes).

## Graphical abstract

Rātā Moehau (Metrosideros bartlettii), a critically endangered tree endemic to Aotearoa New Zealand, is represented by only 14 wild individuals. We assembled its first genome, analysed genetic diversity in wild and cultivated individuals and found long‐term population decline and reduced diversity in cultivated plants—providing key insights to support restoration efforts led by Ngāti Kuri, the local Māori iwi (tribe).

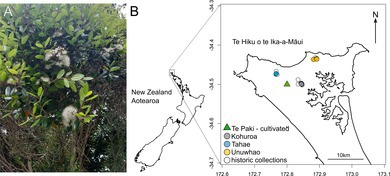

## 1 Introduction

Worldwide a third of tree species are threatened with extinction (Rivers et al. 2023), underscoring the urgent need for targeted conservation efforts. Trees are foundational to terrestrial ecosystems, providing habitat, food and crucial ecological functions such as carbon storage and soil stabilisation (Rivers et al. 2023). However, both habitat fragmentation and small population size increases extinction risk for many species (Ellstrand and Elam 1993; Matthies et al. 2004). In small populations within fragmented habitats, the effects of demographic, environmental and genetic stochasticity are amplified. Genetic drift can lead to a reduction in genetic diversity, while inbreeding and the accumulation of deleterious mutations can reduce fitness (Young et al. 1996).

Understanding both the current genetic diversity and its spatial partitioning, along with the demographic history of a threatened tree species, is essential for developing an effective conservation management plan (e.g., the yellow‐footed rock‐wallaby; Potter et al. 2020). Demographic modelling can help clarify whether a species with a restricted geographical distribution has undergone significant historical decline or if it is in the early stages of speciation and expansion (Li and Durbin 2011).

**FIGURE 1:**
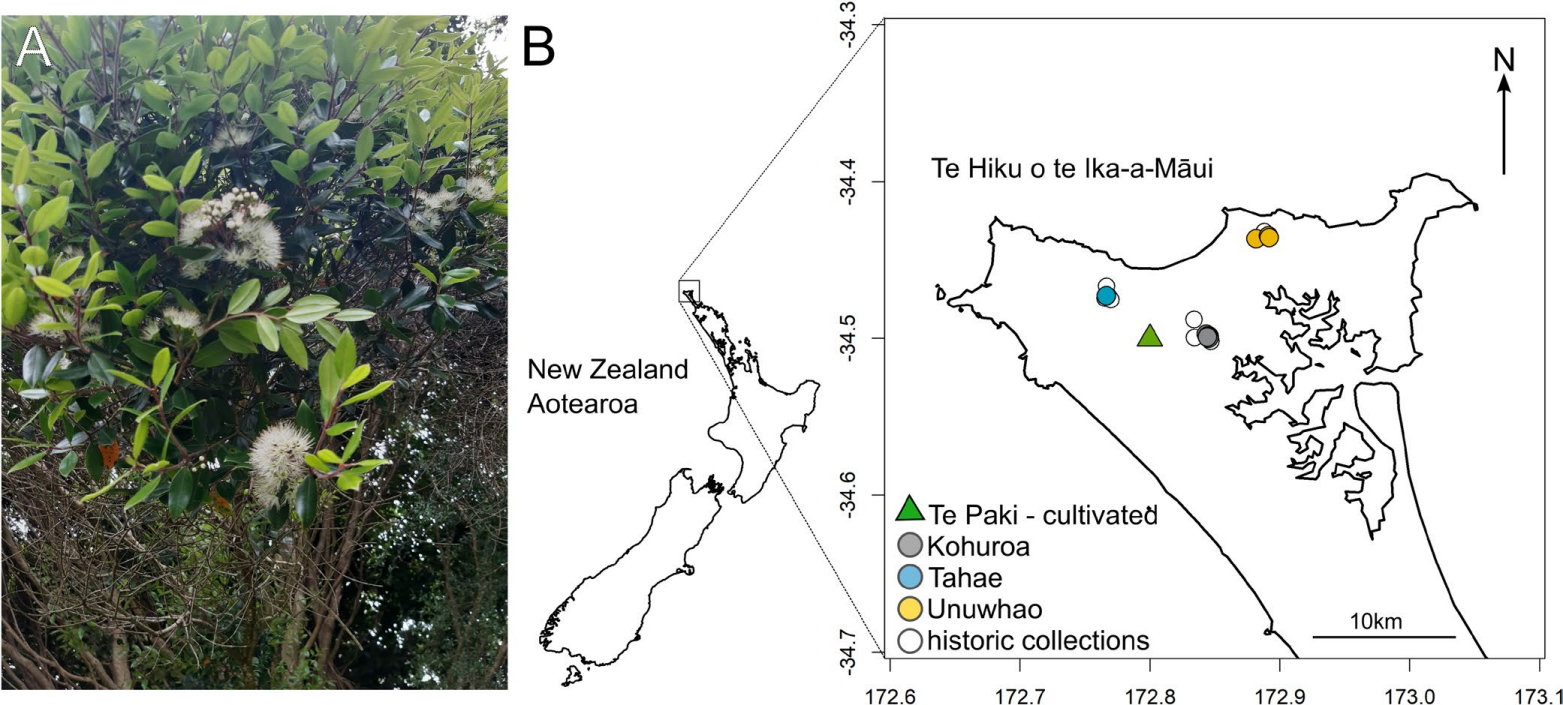
Rātā Moehau photo and distribution. (A) Photograph of a rātā Moehau in flower, this is the individual growing at the Auckland Botanic Gardens that the genome assembly is based on (ABG 19910062). Photograph by Emma Simpkins (nee Bodley). (B) Map of New Zealand and of the northern tip of the North Island of New Zealand showing the current distribution of wild rātā Moehau (coloured circles) compared to historic herbarium collections (white filled circles). The green triangle indicates the Ngāti Kuri nursery at Te Paki.

Rātā Moehau (*Metrosideros bartlettii,* J.W.Dawson, Myrtaceae, Figure 1A) is a tree species endemic to Aotearoa New Zealand (Aotearoa), with its natural distribution confined to Te Hiku o te Ika‐a‐Māui, the northernmost region of Northland (Dawson and Lucas 2011). It faces significant threats to its survival, including habitat destruction, low recruitment and the recent arrival of myrtle rust (*Austropuccinia psidii*). Understanding its genetic diversity and demographic trends is therefore critical for informing conservation strategies and preventing further decline. Rātā Moehau is currently classified as Critically Endangered under the NZ Threat classification System (de Lange et al. 2024) with qualifiers CD (conservation dependent), RR (range restricted) and RF (recruitment failure).

A taonga (treasure) of the iwi (tribe) Ngāti Kuri, rātā Moehau is named in honour of their ancestress, Moehau (Ringham 2022). Rātā Moehau was first described in the scientific literature in 1985 (Dawson 1985), with only seven plants recorded from two localities. Subsequent surveys identified a third location (see Figure 1B), and by 2000, 31 plants were recorded (Drummond et al. 2000). However, more recent surveys indicate a significant decline, and by 2015, only 14 trees were found in the wild (de Lange 2016). The three locations are separated from one another only by kilometres, with Tahae and Kohuroa approximately 7.7 km apart, Tahae and Unuwhao approximately 12 km apart, and Unuwhao and Kuhuroa approximately 8.5 km apart (distances calculated using the tool at https://www.movable‐type.co.uk/scripts/latlong.html, accessed 9 Feb 2026). Using the GeoCAT tool developed by Kew's Spatial Analysis team (http://geocat.kew.org/editor, accessed 9 Feb 2026), the species' Extent of Occurrence is estimated at 36.855 km^2^, and the current Area of Occupancy is estimated at 5.976 km^2^, supporting its Critically Endangered threat listing. Plants of this species have been in cultivation since the 1970s, but the provenance of these plants is not always well known, making it difficult to incorporate them into restoration projects.

Phylogenetic studies using nuclear ribosomal ITS and ETS markers place rātā Moehau as sister to a clade that includes several endemic *Metrosideros* tree species (i.e., pōhutukawa/kahikā *M. excelsa* and *M. robusta* ), as well as *Metrosideros* native to Lord Howe Islands, Rangitāhua/the Kermadec Islands (kahikā Rangitāhua, *M. kermadecensis* ) and East Polynesia (Wright et al. 2021). When considering the plastome marker *trnL–trnF*, rātā Moehau is the only Aotearoa tree *Metrosideros* to not share haplotypes with other species, but this may be due to limited sampling (Gardner et al. 2004). Based on the pollination systems of close relatives, these trees are likely bird‐ and insect‐pollinated (pers. obs.) with distinctive white flowers and white bark. Rātā Moehau is considered highly self‐incompatible, requiring cross‐pollination to produce viable seed (Van Der Walt et al. 2023). They often start life as epiphytes and can grow up to 30 m tall (de Lange et al. 2010).

Previous population genetic studies of rātā Moehau using Amplified Fragment Length Polymorphisms (AFLPs) and microsatellite markers (Drummond et al. 2000; Melesse 2017) reported relatively high levels of heterozygosity despite the species' small census size. Drummond et al. (2000) noted that such levels of diversity are consistent with expectations for long‐lived, outcrossing tree species, which often retain substantial genetic variation even during demographic decline (e.g., Hamrick and Godt 1996; Young et al. 1996). Due to its taonga status and the species' declining numbers, the iwi of Ngāti Kuri are leading a conservation program for rātā Moehau in partnership with scientists. This study aims to support Ngāti Kuri's efforts by providing additional insights for their restoration plan. Specifically, this study aims to:

1. assemble and annotate a reference genome for rātā Moehau,
2. characterise genome‐wide genetic diversity and its spatial distribution across the remaining wild populations using resequencing—a genome‐wide approach that complements earlier microsatellite and AFLP studies (Drummond et al. 2000; Melesse 2017) by sampling a substantially larger portion of the genome, and
3. infer the species' demographic history using whole‐genome resequencing data to test whether the species' current rarity reflects long‐term demographic decline, persistent small population size associated with historical isolation or more recent fragmentation. We also include a small number of samples of cultivated rātā Moehau trees to determine the importance of the cultivated trees for restoration of this species.

## 2 Methods

### 2.1 Sampling

A single tree of rātā Moehau growing at the Auckland Botanic Gardens (ABG; −37.012333, 174.905992) was selected as the source for genome sequencing (Figure 1A). The specimen tree with ABG accession number 19910062 growing in garden bed NZT001 was six meters tall and 3 years old at the time of collecting, on 15th November 2021. Based on prior research, this plant is thought to have originated from Tahae (Radar Bush, Te Paki; Melesse 2017). Then curator of the Native Plant collection, Emma Simpkins (nee Bodley), collected leaf (new season's growth) and flower samples after consultation with Ngāti Kuri, and the samples were transported directly to the −80°C freezer at the Bioeconomy Science Institute, Auckland. A voucher specimen for this tree is now housed at the Allan Herbarium, Bioeconomy Science Institute, Lincoln, accession number CHR 674218 (Specimen Details).

The population genetic inferences for the species were made using leaf tissue from 12 rātā Moehau individuals, 9 wild and 3 cultivated trees (Figure 1B, see Table A1). Tissues from the wild samples were collected in 2015 by the Department of Conservation (Melesse 2017) and stored at −80°C at the Bioeconomy Science Institute, Lincoln.

### 2.2 DNA Extraction and Genomic Sequencing

Using 1 g of rātā Moehau leaf tissue as the starting material, the nuclei were initially isolated following Hilario (2019), though modifying the protocol in that after adding 7 mL of Nuclei Extraction Buffer A, the percoll gradient step was excluded. DNA was then extracted from one of the tubes following the method for extracting high molecular weight DNA from plant nuclei using the Nanobind kit (PacBio, Menlo Park, CA).

Following the nuclei isolation method for DNA extraction, the DNA was sheared by passing it through a 26G needle five times, followed by short fragment removal using the Short Read Eliminator XL kit (PacBio). DNA was then prepared for long‐read sequencing using the ONT SQK‐LSK110 Ligation Sequencing Kit (Oxford Nanopore Technologies, UK). Two libraries were produced and each sequenced independently on an ONT MinION R9.4.1 flowcell for 72 and 68 h, respectively, with flow cells washed and reloaded every 24 h. An Illumina short‐read library was prepared by Livestock Improvement Corporation (LIC, Hamilton, https://www.lic.co.nz/) from the extracted DNA for use in genome polishing and alignment, using the Illumina PCR‐free Library Prep Kit (Illumina, San Diego, CA) with Illumina unique dual indexes ligated to facilitate sample pooling. This was sequenced as part of a larger library pool of similar libraries for other species on an Illumina NovaSeq SP300, 2 × 150 bp.

Two tubes of isolated nuclei prepared in initial DNA extraction were used as input for preparation of a Dovetail Omni‐C library (CantataBio, Scotts Valley, CA), following the Omni‐C Proximity Ligation Assay Non‐mammalian Samples Protocol v1.2 for plants. A total of 1500 ng of DNA was used as input for proximity ligation. Initial testing produced libraries below the desired threshold for total DNA, and so, we increased the input DNA for library preparation from 150 ng to 240 ng, and scaled up all reagents accordingly. A QC run of the final library was sequenced on an Illumina iSeq 2 × 250 bp and assessed for quality using the Phase Genomics hic_qc pipeline prior to full sequencing on an Illumina NovaSeq SP 300 with 2 × 250 bp sequencing at LIC. Both sequencing sets were combined for genome scaffolding following data filtering and trimming.

DNA extractions for the demographic and population‐level analyses were performed using the NucleoSpin Plant II DNA extraction kit (Macherey Nagel, Düren, Germany), with lysis buffer PL2. Samples were prepared for sequencing at LIC, using the low input Illumina PCR‐Free library Prep kit (https://www.illumina.com/products/by‐type/sequencing‐kits/library‐prep‐kits/dna‐pcr‐free‐prep.html). Libraries were sequenced on an Illumina NovaSeq SP (2 × 150 bp) to an average 40× coverage per sample.

### 2.3 RNA Extraction and Sequencing

RNA was independently isolated from leaf and flower tissue from the genome assembly individual with tissue manually ground to a fine powder on liquid nitrogen, using the Modified CTAB Protocol and the Zymo Research Direct‐zol RNA Miniprep kit (Zymo Research, Irvine, CA). Purified RNA was assessed using the Qubit RNA HS Assay kit (Q32852, Thermofisher Scientific, Waltham, MA) and the Fragment Analyser with the High Sensitivity RNA kit (Agilent Technologies, Santa Clara, CA). RNA libraries were prepared and sequenced by the Auckland Genomics Centre (The University of Auckland, Auckland, NZ), following the Illumina Stranded mRNA library prep method, with 500 ng total RNA as input for each sample. Libraries for rātā Moehau were normalised and pooled, and sequenced as part of a larger sample pool on an Illumina Novaseq 6000 with 2 × 150 sequencing.

### 2.4 Genome Assembly

Illumina short‐read data was processed with the TrimGalore v0.6.7 wrapper (https://github.com/FelixKrueger/TrimGalore Krueger 2012) for Cutadapt v3.5 and FastQC v0.11.9 in paired‐end mode, with two‐colour sequencing chemistry specified, minimum Q28, a minimum read length of 50 bp, and 3′ and 5′ clipping. This data was initially used for k‐mer counting with Jellyfish v2.3.0 (Zhang et al. 2014) to estimate genome profiles using GenomeScope (Vurture et al. 2017). ONT long‐reads were basecalled with ONT's Guppy v6.2.1 using the dna_r9.4.1_450bps_sup configuration. Data from the sequencing summary was visualised with Nanoplot (https://github.com/wdecoster/NanoPlot). Raw basecalled sequences passing the default thresholds were assessed with NanoQC v0.9.4 (De Coster et al. 2018) prior to trimming and filtering with Porechop v0.2.4 (https://github.com/rrwick/Porechop) with the *‐‐discard‐middle* flag, and NanoFilt v2.6.0 (De Coster et al. 2018) with minimum length of 500 bp, minimum average quality of 10 and the first and last 20 bp of each read trimmed. Processed data from the two sequencing runs was combined and assembled with Shasta v0.10.0 (https://github.com/paoloshasta/shasta). Following each step in the assembly process, genome assembly summary metrics were assessed, including representation of BUSCO orthologues from the eudicots_odb10 database using Compleasm v0.2.2 (Huang and Li 2023) and k‐mer evaluation with Merqury v1.3 (Rhie et al. 2020). The final assembly was assessed with Blobtoolkit (Challis et al. 2020).

The purge_dups pipeline (Guan et al. 2020) was used to remove overlaps with Minimap2 v2.24 (Li 2018). Two rounds of polishing were conducted with Racon v1.5.0 (https://github.com/lbcb‐sci/racon) with the processed ONT data, followed by one round with Medaka v1.11.1 (https://github.com/nanoporetech/medaka) using the r941_min_sup_g507 model, and finally, two rounds with NextPolish v1.4.1 (Hu et al. 2020), implementing SAMtools v1.13 (Danecek et al. 2021) and BWA v0.7.17 (Li and Durbin 2009) for mapping the processed Illumina short‐read data.

The Omni‐C data were processed (adapters removed, Q > 20, length ≥ 50 bp, 15 bp front and tail trimming) with Fastp v0.23.2 (Chen et al. 2018). The polished contigs were then used as the base for scaffolding following the Dovetail Omni‐C pipeline, implementing BWA, SAMtools v1.15.1 and Pairtools v1.0.2 (Open2C et al. 2024) for mapping, filtering and sorting steps. The resulting data were passed to YaHS v1.2a.2 (Zhou et al. 2023) with the *‐‐no‐mem‐check* flag, completing eight iterations of scaffolding. The scaffolded assembly was screened for contamination using NCBI's FCS‐GX pipeline (Astashyn et al. 2024). Scaffolds with non‐family or organellar hits were excluded from the assembly.

### 2.5 Genome Annotation

#### 2.5.1 Repeat Annotation

We first performed repeat annotation of the assembled genome. RepeatModeler v2.0.3 (Flynn et al. 2020) was used to build a repeat library. An iterative process was used to classify those ‘unknown’ repeats identified with RepeatModeler using the *repclassifier* tool in RepeatMasker v4.1.0 (Smit and Hubley 2025) using the Eukaryota elements from RepBase, until no additional unknown repeats were classified. RepeatMasker was then used to annotate (a) simple repeats, (b) complex repeats, (c) known repetitive elements from the previously constructed repeat library and (d) remaining unknown repetitive elements from the same repeat library. The annotations from each stage were then combined and summarised with RepeatModeler's *ProcessRepeats* tool. We used *rmOutToGFF3custom* (Card 2024) to convert the combined, simple and complex outputs to GFF3 format. The combined simple and complex repeat annotations were used to produce a soft‐masked version of the assembly that was passed as input for gene annotation.

#### 2.5.2 Gene Annotation

Raw RNA‐seq data were processed with TrimGalore to remove adapters and perform quality trimming (−*q* 20). Trimmed reads were then screened for contamination with Kraken2 v2.1.3 (Wood et al. 2019), and outputs were processed with Bracken v2.7 (Lu et al. 2017) using the standard Kraken database downloaded June 2024. Reads retained following screening were passed to SortMeRNA v4.3.6 (Kopylova et al. 2012) to exclude reads originating from rRNAs.

The soft‐masked reference assembly was indexed with STAR v2.7.10b (Dobin et al. 2013) for use as the reference for transcriptome annotation. The processed RNA‐seq reads were independently aligned to this reference with STAR with readgroup attributes set manually, with *‐‐outSAMstrandField intronMotif* set and to produce unsorted BAM files. These alignments were then sorted with SAMtools v1.19, and alignment summary statistics were obtained with Picard v2.26.10 (Broad Institute 2019).

We supplemented the RNA‐seq annotation with the OrthoDB Viridiplantae protein database (downloaded 2024‐09‐05). Sequences containing non‐alphabetical characters were removed, and the database was processed for use with the reference assembly using BRAKER v3.0.8 (Gabriel et al. 2024) *prothint.py*. We passed the RNA‐seq alignments to BRAKER, with BUSCO lineage set to eudicots_odb10. We independently performed annotation with BRAKER using the OrthoDB protein hints. Computational challenges prevented the use of the full BRAKER3 pipeline, so after identifying the best quality annotation output of each independent BRAKER run, we merged the Augustus and GeneMark‐ET gene prediction outputs and the extrinsic evidence hints files from the respective annotations using TSEBRA v1.1.2.5 (Gabriel et al. 2021).

Post‐processing of the merged annotation involved screening the output to exclude overlapping genes, retain only the longest isoforms and remove annotations lacking start or stop codons with AGAT v1.0 (Dainat et al. 2024). AGAT was also used to collect additional statistics from the filtered GFF and to convert this to FASTA format. The protein sequence FASTA was assessed for completeness against the eudicots_odb10 with compleasm v0.2.6 in protein mode. We ran all vs. all sequence alignments with BLAST v2.16.0 to confirm that transposable elements in the reference assembly had been adequately masked.

We used *blastp* in DIAMOND v2.1.10 (Buchfink et al. 2021) to align annotated proteins to gene models in the UniProtKB database in sensitive mode with a maximum e‐value of 0.00001. The hit with the highest bitscore was retained for each transcript sequence and then UniProt accession IDs were passed to the UniProt ID‐mapping webtool (www.uniprot.org/id‐mapping) to gather functional annotation data (product descriptions and Gene Ontology (GO) terms). We also used InterProScan v5.66–98.0 (Jones et al. 2014) to identify protein domains from the PFAM database and obtain corresponding GO terms. We merged the repeat and gene annotation GFFs and appended the cleaned, combined functional annotation information for transcript sequences.

### 2.6 Population Genetic Diversity and Demographic Analyses

#### 2.6.1 Variant Calling

We assessed read quality using FastQC v0.12.1 (Andrews 2010) and MultiQC v1.13 (Ewels et al. 2016) before trimming reads using TrimGalore v0.6.10 (Krueger 2012). Paired‐end reads were trimmed to a minimum length of 50, with a 3′ clip of 5 bp and a 5′ clip of 20 bp, using a 2‐colour compatible minimum quality score of 20 and a clip of 20 bases. We then mapped the samples using bwa mem and generated mapping stats (BWA v0.7.17 and SAMtools v1.10; (Danecek et al. 2021)).

Variants for each individual were detected following the variant calling method of Magid et al. (2022), using the split_bamfiles_tasks.pl. script to split alignments into smaller chunks prior to calling variants for all individuals against the reference genome with BCFtools v1.15.1 (Li 2011; Danecek et al. 2021) *mpileup* and *call*. The outputs were then recombined into a single coordinate‐sorted variant file, and variants were filtered to extract high‐quality biallelic SNPs. We trialled multiple settings for SNP filtering including missing data set to 0, 0.1, and 0.25; minimum coverage of 10×, 15× or 20×; linkage disequilibrium of 0.8, 0.6 or 0.4; and minor allele frequency of 0, 0.05 and 0.1.

#### 2.6.2 Population Genetic Clustering and Summary Statistics

K‐means clustering was used to group individuals using the *find.clusters* function from adegenet v2.1.10 in R v4.4.1(Jombart 2008). The analysis was performed with the maximum number of clusters (*max.n*) set at 10 and the number of principal components (*n.pca*) set to the number of individuals in the analysis (i.e., 12). Choice of the optimal number of principal components was made interactively based on Bayesian Information Criterion (BIC) score. Clustering was also explored using FastStructure v1.0 (Raj et al. 2014) which implements a Bayesian approach. We also performed principal component analysis (PCA) using the *snpgdsPCA* function from the SNPRelate v1.38.0 package (Zheng et al. 2012) in R and network analysis based on the Euclidian distance using SplitsTree v4 (Huson and Bryant 2006) to further assess structure in the data. Expected and observed heterozygosities and F_IS_ was calculated from the outputs of VCFftools ‐*‐het* and visualised in R.

#### 2.6.3 Demographic Analysis

Demographic analysis was conducted using the Pairwise Sequentially Markovian Coalescent (PSMC) model (PSMC v0.6.5). Originally developed for use in humans (Li and Durbin 2011), PSMC has since been applied to a variety of other organisms, including walnuts (*Juglans* spp., (Bai et al. 2018)). This method infers changes in effective population size (Ne) over time from a single diploid genome sequence. We generated a consensus sequence for one wild resequenced individual (individual 17, from Tahae; see Table A1 in Appendix 1 for voucher details) and converted it to PSMC input format using the *fq2psmcfa* command from the PSMC package, with a quality cut‐off of 10 (−*q* 10).

Demographic inference was performed using the *psmc* command with the following parameters: the maximum number of iterations (−*N*) was set to 50; the maximum time (−*t*) was tested with values 5, 10 and 15; the initial mutation/recombination ratio (−*r*) was set to 5; and the atomic time interval pattern (−*p*) was set to ‘4 + 25*2 + 4 + 6’, based on PSMC guidelines. All parameter sets converged when Tmax was set to 5 or 10 but not 15, so we continued using –*t* 10.

To appropriately scale the inferred demographic history, we tested several combinations of generation time and mutation rate. We evaluated four different mutation rates (Table 1): two derived from experimental data ( *Arabidopsis thaliana* , (Ossowski et al. 2010); *Prunus* spp., (Xie et al. 2016)), and two estimated using fossil or other calibration methods (Brassicaceae, (De La Torre et al. 2017); *Juglans* spp., (Bai et al. 2018)). Although mutation rates for other angiosperm families have also been estimated using fossil calibrations (as summarised in (De La Torre et al. 2017)), we selected the highest and lowest rates identified to capture the full range of plausible values.

**TABLE 1:** The different mutation rates assessed for scaling the PSMC plots.

Study focus	References	Mutation rate per site per year	Mutation rate per site per generation	Mutation rate, G = 20
Brassicaceae	De La Torre et al. 2017	6.52 × 10^−9^		1.304 × 10^−7^
Walnut (*Juglans*)	Bai et al. 2018	2.06 × 10^−9^		4.12 × 10^−8^
*Arabidopsis* ^a^	Ossowski et al. 2010		7.0 × 10^−9^	7.0 × 10^−9^
Peach (*Prunus*)^a^	Xie et al. 2016		9.5 × 10^−9^	9.5 × 10^−9^

*Note:* The mutation rates with generation time of 20 years were those applied for rātā Moehau.

Abbreviation: G, generation time in years.

^a^ Used by Choi et al. (2021) for Hawaiian *Metrosideros*.

Selecting an appropriate generation time for a long‐lived tree species such as rātā Moehau is not straightforward. A plant grown from a cutting took 25 years to flower (Lehnebach 2017; Nadarajan et al. 2021), but little additional information is available regarding the typical age at first flowering. These trees also do not appear to flower annually; in cultivation it has been noted that flowering typically occurs every four years (Bodley and Stanley 2019). Lack of regular flowering was also indicated in the formal scientific description of the species (Dawson 1985), where it is noted that although the tree was first recognised as distinct in 1977, flowering was not observed until 1984. The longevity of these trees is also unknown, but the congeneric and closely related *Metrosideros excelsa* may be able to live for up to 1000 years (Simpson 1994).

Given this limited information, we trialled the generation time used by Choi et al. (2021) for Hawaiian *Metrosideros* (20) across all four mutation rates. Additionally, we tested a broader range of generation times (G = 1, 2, 5, 10, 20, 30 and 100 years) using the *Juglans* mutation rate, based on its similar life history traits to rātā Moehau as a long‐lived tree species.

## 3 Results

### 3.1 Genomic Sequencing

ONT sequencing of two libraries produced 2.57 million reads totalling 43.8 Gb raw data, for approximately 125× coverage based on an estimated 350 Mb genome size (Table A2 in Appendix 1). Mean read length of the first library was 14 kb, while the mean length was 21 kb for the second. The combined Omni‐C sequencing outputs produced 51.6 Gb data, for approximately 172× coverage. Illumina short‐read sequencing for QC and polishing produced approximately 49× coverage.

Sequencing of the two RNA‐seq libraries produced 83.1 million reads totalling 12.5 Gb of data. Following processing, 75.97 million reads were retained (91.4%) for alignment to the assembly. Of the retained reads, 17.4 million were successfully aligned.

### 3.2 Genome Assembly and Annotation

**FIGURE 2:**
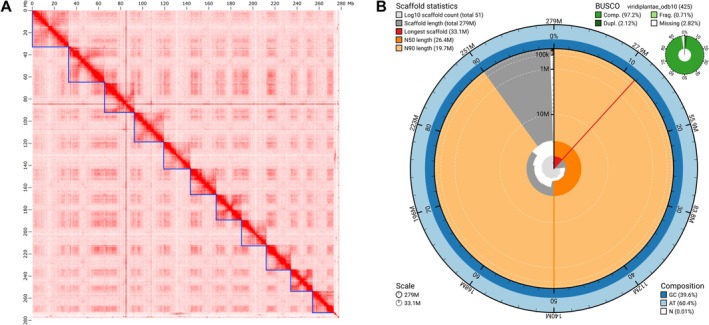
Graphical representation of the Rātā Moehau genome assembly. (A) Juicebox Hi‐C contact map from after manual curation; Mb = megabases. (B) snail plot of final assembly metrics, including compleasm results for Viridiplantae BUSCOs (*n* = 425); M = megabases. BUSCO results: Comp. = complete, Dupl. = duplicated, Frag. = fragmented.

High‐quality long‐read data in combination with Omni‐C data produced a highly contiguous and complete assembly for rātā Moehau, at 279.3 Mb forming 11 super‐scaffolds representing the expected 11 chromosomes (Figure 2). This was close to the expected genome size of 256 Mb estimated from k‐mer analysis of short‐read data with GenomeScope. N50 scaffold length was 26.4 Mb (Table 2). The assembly achieved 96.0% BUSCO completeness.

**TABLE 2:** Genome assembly and annotation metrics for rātā Moehau.

**Assembly metrics**	
Number of scaffolds	51
Genome size (bp)	279,317,961
Scaffold N50 (bp)	26,431,966
Scaffold L50	5
Longest scaffold	33,083,982
**Annotation results**	
mRNAs	5813
Exons	123,888
CDS	123,888
Introns	94,102
Genes	29,786
Start codons	29,785
Stop codons	29,785
Transcripts	29,786

**Completeness**	**Assembly**	**Annotation**
Complete	2233 (96.0%)	2155 (92.6%)
Complete single copy	2200 (94.6%)	1895 (81.5%)
Complete duplicated	33 (1.4%)	260 (11.2%)
Fragmented	10 (0.4%)	17 (0.7%)
Missing	83 (3.6%)	154 (6.6%)
Total assessed	2326	2326

*Note:* The BUSCO annotation values are those from the TSEBRA merged annotation output. BUSCO completeness was assessed via Compleasm v0.2.5 for the assembly and v0.2.6 for the annotation.

Abbreviations: bp, base pairs; CDS, coding DNA sequences; Gb, gigabases; MRNAs, messenger RNAs.

RNA sequencing of leaf and flower tissue produced 83 million paired‐end reads, totalling 12.46 Gb. Repeat annotation identified 37.1% of the assembly as repetitive elements. These repetitive elements were primarily unclassified elements (8.7%) or long terminal repeats (7.5% Ty3, 5.8% Caulimovirus and 5.5% Copia; see Table A3 in Appendix 1). The filtered output of the TSEBRA merging process contained 29,786 genes, with 92.6% BUSCO protein completeness for eudicots (Table 2). Average gene length was 2413 bp, with 25.7% of the genome composed of genes. Functional annotation of transcripts via DIAMOND assigned UniProt accession IDs for 20,028 transcripts (67.2%), similar to functional annotation via InterProScan, which assigned PFAM IDs for 19,960 transcripts (67.0%). UniProt and PFAM IDs, product descriptions and GO terms were added to these transcript records in the final GFF.

### 3.3 Low‐Coverage Whole Genome Resequencing

An average of 93.7 million reads were generated for each of 12 samples, ranging from 59.6 million to 120.6 million (Table 3). The sequences were of high quality, with quality scores higher than 35 for most of the length of each read and only an average of 1.4 million reads were trimmed for each sample. After trimming, the level of duplication per sample was on average 19.3%. An average of 90.55% of sequences mapped to our assembled genome, resulting in an average coverage of 45× (using our newly calculated genome size of 279.3 Mb).

**TABLE 3:** Description of sequencing outputs and genetic diversity metrics for rātā Moehau samples.

Sample ID	Population	Reads (M)	Reads retained (M)	Duplication in retained data (%)	Mapped reads (%)	Average coverage	H_O_	H_E_
JF0657	Cultivated, origins unknown	120.6	117.8	21.98	95.22	60.7	0.38	0.37
BV0767	Cultivated, origins unknown	116	114	30.53	93.61	58.0	0.35	0.37
20170833	Cultivated, cutting from Unuwhao	86	84.8	20.95	94.44	43.5	0.46	0.37
3	Unuwhao	87.8	86.6	19.80	91.42	43.0	0.49	0.37
5	Unuwhao	97.2	96	19.40	90.06	46.9	0.49	0.37
12	Kohuroa	99.8	98.2	16.65	80.19	42.7	0.49	0.37
16	Kohuroa	84.2	83.2	17.85	93.01	41.9	0.52	0.37
17	Tahae	95.2	94	18.60	92.48	47.1	0.44	0.37
270	Unuwhao	94.2	92.8	15.38	78.17	39.2	0.49	0.37
271	Unuwhao	88.2	87.2	17.53	93.15	44.1	0.46	0.37
281	Kohuroa	95.6	94.4	18.55	91.31	46.7	0.49	0.37
286	Kohuroa	59.6	59.2	13.53	93.53	30.0	0.46	0.37
Mean		93.7	92.4	19.23	90.55	45.3	0.46	0.37

*Note:* Retained data refers to those reads retained following trimming and filtering.

Abbreviations: H_E_, expected heterozygosity; H_O_, observed heterozygosity; M, million.

### 3.4 Variant Calling

Trialling multiple settings for SNP filtering led to 156 different SNP datasets, ranging from the smallest with 2080 SNPs when set to allow no missing data, 20× minimum coverage, pruned for linkage disequilibrium with *r* ^2^ = 0.4 and minor allele frequency of 0.25. The largest dataset contained 6,601,731 SNPs when set to 0.25 missing data, 10× minimum coverage, *r* ^2^ = 1.0 and minor allele frequency of 0. Our final filtering strategy retained biallelic SNPs with a minimum coverage of 20×, no filtering for minor allele frequency, *r* ^2^ = 0.8 and no missing data, producing a dataset of 114,478 SNPs.

### 3.5 Population Genetic Clustering and Summary Statistics

**FIGURE 3:**
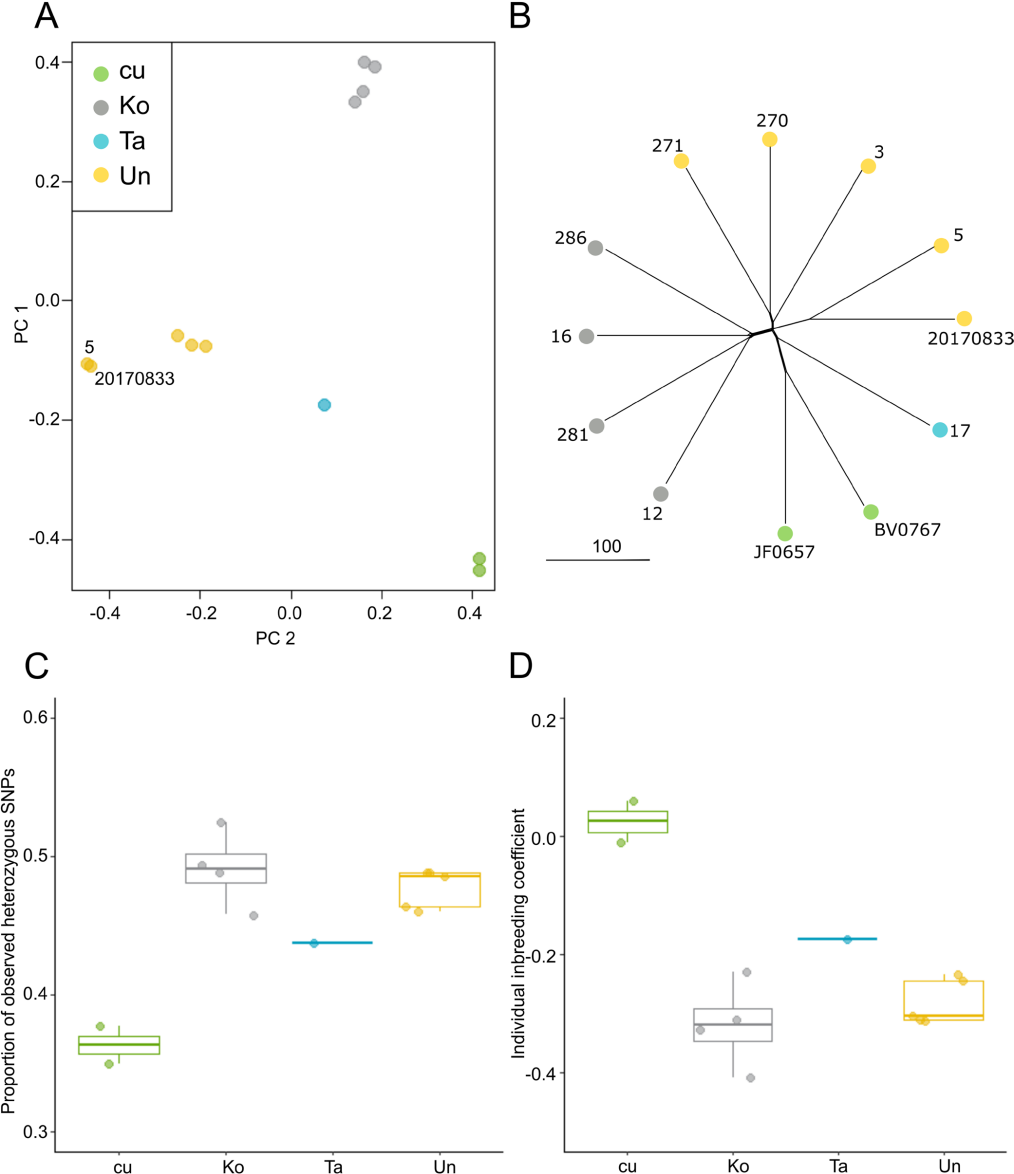
Results of the rātā Moehau population genomic analyses. (A) Principal Component Analysis (PCA) to assess potential genetic structuring across the geographic distribution. (B) NeighborNet network, with individual sample names. C. Observed heterozygosity by location, and D. Inbreeding coefficients (F_IS_) by location. Cu = cultivated, Ko = Kohuroa, Ta = Tahae, Un = Unuwhao.

Principal component analysis (PCA) revealed genetic differentiation between samples, with individuals from the same geographical locations clustering together (Figure 3A). However, the optimal number of genetic clusters was determined to be one by both K‐means clustering and FastStructure, suggesting that all individuals are genetically very similar (data not shown). Notably, one cultivated sample (20170833‐ABG) clustered tightly with a wild individual from Unuwhao (5‐Unuwhao). This is predictable as the cultivated sample is grown from a cutting from that wild individual (see voucher information in Table A1 in Appendix 1). In contrast, network analysis highlighted differences between these duplicate samples, likely due to allele dropout during SNP calling (Figure 3B).

Observed heterozygosity was higher than expected in all wild individuals, whereas the two cultivated plants showed observed heterozygosity consistent with expectations (Table 3, Figure 3C). Mean observed heterozygosity was 0.479 for wild individuals compared with 0.365 for cultivated individuals (expected heterozygosity = 0.37 for all). Similarly, inbreeding coefficients (F_IS_) were negative for the wild individuals, indicating lower relatedness than expected under random mating, while the cultivated plants had F_IS_ values near zero or slightly negative, consistent with low levels of inbreeding (Figure 3D).

### 3.6 Demographic Analyses

**FIGURE 4:**
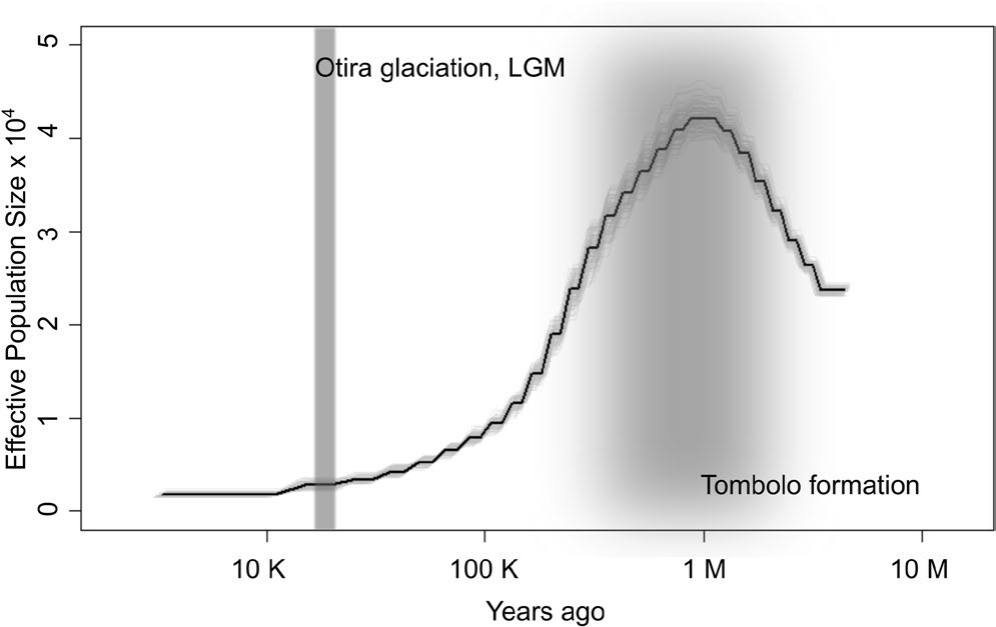
Demographic modelling for rātā Moehau using PSMC. Generation time of 20 years, mutation rate of 4.12 × 10^−8^. Grey vertical bars indicating approximate dates for key events, i.e., the tombolo formation during the middle of the Pleistocene, and the last glacial maxima (LGM) approximately 18,000 years ago.

We selected a generation time of 20 years and mutation rate of 4.12 × 10^−8^ as the most appropriate for rātā Moehau. The PSMC analyses with these parameters show a peak in effective population size (*Ne*), which declines steadily toward the present (Figure 4). The four different mutation rates affect both the timing and magnitude of this peak: the highest mutation rate results in a smaller, more recent peak, while the lowest mutation rate produces the largest and oldest peak. For the two mutation rates defined per generation (*Prunus* and *Arabidopsis*), altering the generation time shifts the entire curve backward in time. In contrast, for the two mutation rates defined per year, the rate must be recalculated when generation time changes—this results in a lower peak at the same time point when generation time increases.

## 4 Discussion

In this study, we developed new genomic resources for rātā Moehau, a species of high conservation concern. This includes the first reference genome for the species, providing a foundation for population‐level genomic characterisation. Using genomic resequencing data, we estimated genome‐wide diversity and compared genetic variation between the few remaining wild individuals and a subset of cultivated individuals. These comparisons revealed reduced genetic diversity among the cultivated population. Demographic modelling indicates a significant decline in population size beginning approximately one million years ago. Together, these findings have important implications for conservation and restoration planning.

### 4.1 Genome Assembly and Annotation

The vast majority of Myrtaceae genome assemblies available at the time of writing were for eucalypts (60%; see Table S1); therefore, the rātā Moehau assembly presents a valuable contribution to the genome resources for this family as only the second assembly available for *Metrosideros* spp. after *Metrosideros polymorpha var. incana* (Choi et al. 2021). The rātā Moehau assembly consists of 11 superscaffolds representing chromosomes, consistent with existing Myrtaceae genome assemblies (e.g., *Eucalyptus grandis* , NCBI genome accession GCF_016545825.1, *Syzygium malaccense*, genome accession GCA_031216405.1; see Table S1). Among the assembled Myrtaceae genomes, genome size ranged from 187 Mb to 1.1 Gb, averaging 499 Mb. At 279 Mb, the rātā Moehau assembly is one of the smaller Myrtaceae genomes assembled to date, similar to that of close relative *Metrosideros polymorpha var. incana* at 284 Mb.

We observed a high degree of duplication in the TSEBRA merged annotation for rātā Moehau, likely due to the merging resulting in inconsistencies due to overlapping gene regions annotated, or isoforms, but this was resolved through subsequent filtering steps. The final annotation consisting of 29,786 protein‐coding genes is within the expected number of genes for Myrtaceae (21,240–42,619 genes annotated; for example, *Psidium guajava* , genome accession GCA_023344035.1; *Eucalyptus grandis* , genome accession GCF_016545825.1; see Table S1).

### 4.2 Population Genomic Diversity and Differentiation

All of the rātā Moehau individuals included in this study were found to be very closely related, with cluster analyses supporting a single genetic grouping across samples. The separation observed in the PCA should be interpreted in this context. The PCA shows distinct grouping of the three wild populations and closely associates the two duplicate samples. The two cultivated individuals are not closely aligned with any wild population, though they are positioned nearest to the wild sample from Tahae, suggesting they are most likely descendants of cuttings taken from that location. Similarly, the NeighborNet network places the two cultivated samples on a branch shared with the Tahae sample, further supporting this interpretation. This is consistent with records indicating that all cultivated material likely originates from either Tahae or Kohuroa (as Kohuroanaki; de Lange 2016; Melesse 2017).

The expected and observed heterozygosity was relatively high for all individuals included in this study, for example in comparison to the related *Eucalyptus* (Silva‐Junior et al. 2015). Although as noted in the introduction, relatively high heterozygosity is to be expected for a long‐lived outcrossing tree species (Young et al. 1996). These high values therefore may be due to the breeding system and longevity of rātā Moehau trees, as well as potentially reflecting a larger effective population size in the past. Previous genetic studies focussed on rātā Moehau have included genotyping of AFLPs and microsatellite markers (Drummond et al. 2000; Melesse 2017). Drummond et al.'s (2000) study, including 31 samples, described genetic diversity using the average heterozygosity metric (H) of 0.18. This was interpreted as relatively high compared to other studies at the time, although they noted that comparisons were made with plants of different life history traits. Melesse's 2017 study (Melesse 2017) included 21 samples, though it remains unclear whether these are all separate individuals, as there were only 14 plants reported in the wild at the time (de Lange 2016). Their analysis measured genetic diversity using the expected heterozygosity metric (*H*_E_), which yielded a value of 0.17, interpreted as low compared to other species with similar life history traits. Both values are much lower than that of the present study; however, these values are likely not directly comparable due to differences in marker properties. For example, the present SNP dataset, while covering a higher proportion of the genome, is based only on variable sites. In addition, differences between the mutation rates of the two markers likely make them difficult to compare directly (e.g., Zimmerman et al. 2020).

In terms of spatial structure, Drummond et al. (2000) distinguished the two plants at the Tahae location (called Te Paki or Radar Bush in their study) from the plants at the other two locations (Kohuroa and Unuwhao), which were intermixed, whereas Melesse (2017) separated the plants at the Kohuroa location from plants at the other two locations (Tahae [as Te Paki] and Unuwhao), which were intermixed. In contrast, our genome‐wide SNP data clearly separate all three wild populations.

We observed that the two cultivated rātā Moehau individuals exhibit reduced heterozygosity and increased inbreeding compared to their wild counterparts. These plants were sourced from a commercial nursery, and their provenance (whakapapa) is unknown. In this species, mechanisms such as temporal separation of stigma receptivity and pollen shed (i.e., dichogamy; Lloyd and Webb 1986) help to prevent self‐pollination. However, van der Walt et al. (Van Der Walt et al. 2023) did report on one instance where a tree produced a small number of viable self‐pollinated seeds. It is therefore plausible that the reduced genetic diversity and elevated inbreeding observed in the cultivated individuals reflect a recent selfing event. If multiple plants were propagated from cuttings of the same individual and later crossed, this could similarly result in reduced genetic diversity. This highlights the importance of promoting outcrossing between individuals of known provenance to maintain genetic diversity in wild populations. Introducing individuals of unknown origin from commercial nurseries poses a risk, as they may be inbred and could contribute to reduced genetic diversity and lower overall fitness.

### 4.3 Demographic History and Biogeography

Rātā Moehau is restricted to the Te Haumihi region, at the most northern tip of Aotearoa. This region is rugged hill‐country formed from Cretaceous to recent rock formations (Leitch 1970; Brook 1999). The area is notable for its ultramafic rock units and associated soils, which produce challenging conditions for plant growth. This region is connected to mainland Northland by a long tombolo that formed following at least the Middle Pleistocene (Brook 1999; Hayward 2017). Te Haumihi was therefore an island, isolated from the rest of Aotearoa for at least some of the Pliocene and Pleistocene (Hayward 2017). This isolation, coupled with ecological pressures, such as ultramafic soils, has led to extensive allopatric speciation. Consequently, the region has a high number of invertebrates (e.g., Hoare 2010; Buckley and Bradler 2010; Seldon and Leschen 2011), vertebrates (e.g., Chapple et al. 2008) and endemic plants (e.g., de Lange et al. 2003), of which rātā Moehau is a significant example.

While it can be assumed that many of species currently restricted to Te Hauhimi have only ever inhabited this area, it is possible that some have relictual geographical distributions and were formerly more widespread across Northland. Such species may be characterised by a long‐term decline in population size. Species that have always been restricted to Te Haumihi may have had a relatively constant population size and species that recently underwent speciation may have had an increasing population size in the recent past. Coincident with island and tombolo formation since the Pliocene, there have also been extensive climatic changes with repeated cooling and warming cycles since the Pliocene (Newnham et al. 1999). Climatic changes had dramatic effects on the distribution of tree species (Newnham et al. 2013). While widespread species found on mainland Northland would have been able to retreat to refugia, species endemic to Te Haumihi would have had less opportunity due to their naturally restricted geographic distribution, and population size changes may have been more dramatic.

Despite numerous phylogenetic studies on species endemic to Te Haumihi (Buckley and Leschen 2013; Ball et al. 2024), their demographic history has not been investigated in detail. Our demographic reconstruction points to a much larger effective population size for rātā Moehau approximately 1 million years ago (mid Pleistocene), a steep decline until 100 thousand years ago (late Pleistocene), followed by a steady decline to the present. The sister group to rātā Moehau is a clade of *Metrosideros* species found in New Zealand, the eastern Pacific and as far north as Hawaiʻi (Wright et al. 2021). At what point rātā Moehau separated from this clade is unknown, so the large peak in the mid Pleistocene may reflect a larger ancestral gene pool prior to speciation. It is also important to remember that effective population size estimates can be misleading if migration is occurring (Ryman et al. 2019), so the numbers generated should not be taken literally.

The distribution of forest within Te Haumihi is quite different now to what it was in the past. For example, palynological studies show podocarp trees were likely more widespread in the Quaternary (Dodson et al. 1988; Enright et al. 1988). Today, forest patches are highly restricted, mainly due to deforestation following European arrival in the area in the 19th century and continuing into the 20th century. This deforestation would certainly have placed pressure on the rātā Moehau population size, but such a decline would have occurred within the past 200 years. The decline detected in our analysis is much older, dating back to the mid Pleistocene. This population size reduction occurred during the period of island isolation and reconnection to the mainland via tombolo formation, as well as Pleistocene climatic fluctuations. It is likely that some combination of these events caused a long‐term reduction in the population size of rātā Moehau. The more recent deforestation is unlikely to have left an imprint on the pattern of heterozygosity within and among individuals, due to very recent timescale involved relative to the generation time of this species.

### 4.4 Considerations for Future Conservation of Rātā Moehau

With a clearer understanding of the diversity and demographic history of rātā Moehau, this study provides valuable information to guide future conservation efforts aimed at supporting species recovery. Conservation management decisions will be led by Ngāti Kuri, who hold kaitiakitanga (guardianship) over this taonga (treasure).

Beyond immediate insights into diversity and demographic history, the reference genome provides a foundation for long‐term conservation planning that supports kaitiaki in their role as guardians of this taonga species. By enabling high‐resolution mapping of both neutral and adaptive variation, these genomic resources facilitate monitoring of genetic diversity over time, and identification of individuals with unique or underrepresented alleles, thus informing decision‐making for management such as through propagation, translocation or restoration—ensuring that both current and future populations maintain evolutionary potential (Fuentes‐Pardo and Ruzzante 2017; Aitken et al. 2024; Hogg 2024).

Future conservation strategies may include the propagation of individuals for both wild release and managed cultivation, as well as the preservation of *ex situ* genetic diversity through methods such as seed banking. Based on the findings presented here, we recommend that any conservation approach prioritise maximising genetic diversity and minimising inbreeding. This can be achieved by promoting cross‐pollination between individuals from both wild populations and cultivated stock, but only if their provenance is known. Given their sporadic flowering and the large stature of mature wild plants, this will be a challenge, but can be facilitated by storing pollen under conditions identified previously (Van Der Walt et al. 2023).

Currently, approximately 44,248 seeds are stored in the Margot Forde Seedbank and additional collections from wild individuals could further capture the remaining extant diversity. Careful, long‐term management—including the use of studbooks or similar record‐keeping systems—will be important for tracking lineage, genetic diversity and other key aspects of the species over time.

In addition to decisions around breeding and propagation to support species recovery, broader ecosystem factors must also be considered. Browsing by brush‐tailed possums ( *Trichosurus vulpecula* ) can have significant impacts on seedlings (de Lange et al. 2010), potentially limiting regeneration. Therefore, possum control will be essential within both in situ and ex situ sites.

Additional threats include disease‐causing pathogens such as myrtle rust, which has had major impacts on other Myrtaceae species in Aotearoa New Zealand (Berthon et al. 2018; Black et al. 2019). Climate change, and particularly a trend toward warmer and wetter conditions, may facilitate the spread of such pathogens into new areas. Ongoing monitoring of both disease presence and environmental conditions will be important for early detection and rapid response.

Maintaining healthy pollinator populations will also be critical for natural breeding success. While current knowledge of rātā Moehau's pollinators is limited, they are thought to include a range of native birds and insects. Future research into pollinator diversity and interactions will help to better understand and support these ecological networks. Similarly, assessment of the phylogenetic placement of rātā Moehau among the twelve endemic *Metrosideros* spp. will be beneficial for inference of the biodiversity and ecosystem connections of rātā Moehau.

## Author Contributions

**Jessie M. Prebble:** conceptualization (lead), data curation (equal), formal analysis (equal), investigation (lead), methodology (equal), project administration (lead), visualization (equal), writing – original draft (equal), writing – review and editing (equal). **Natalie J. Forsdick:** conceptualization (lead), data curation (lead), formal analysis (equal), investigation (equal), methodology (equal), visualization (equal), writing – original draft (equal), writing – review and editing (equal). **Duckchul Park:** data curation (supporting), methodology (equal), writing – original draft (supporting), writing – review and editing (supporting). **Alexander J. F. Verry:** methodology (equal), writing – original draft (supporting), writing – review and editing (supporting). **Emma Simpkins:** resources (lead), writing – review and editing (supporting). **Thomas R. Buckley:** conceptualization (lead), funding acquisition (lead), project administration (equal), writing – original draft (equal), writing – review and editing (equal).

## Funding

This research was supported by the Genomics Aotearoa High Quality Genomes and Population Genomics project. Funding was also provided by the New Zealand Ministry of Business, Innovation and Employment's Science and Innovation group Strategic Science Investment Fund for Crown Research Institutes and by the Ministry of Business, Innovation and Employment (Ngā Rākau Taketake—Myrtle Rust and Kauri Dieback Research, C09X1817).

## Conflicts of Interest

The authors declare no conflicts of interest.

## Supporting information


**Table S1:** Genome assembly and annotation information for Myrtaceae with genome assemblies available through NCBI as at 19 May 2025. [Separate supplementary file].

## Data Availability

Rātā Moehau are a taonga (treasure) for the Ngāti Kuri iwi (tribe), and the genomic data derived from these plants are taonga in their own right. Raw and analysed data and associated metadata are available through the Manaaki Whenua—Landcare Research data repository (https://doi.org/10.7931/0sc9‐dp46) with managed access. These data may be made available at the discretion of representatives of Ngāti Kuri, contact research@ngatikuri.iwi.nz to request permission. All scripts associated with bioinformatic analyses are available at our Rātā Moehau GitHub: https://github.com/GenomicsAotearoa/High‐quality‐genomes/tree/main/R%C4%81t%C4%81‐Moehau.

## Appendix 1

**TABLE A1:** Sample table with voucher information for rātā Moehau samples included in the resequencing study.

DNA resequencing ID	Sample ID	Location	Herbarium voucher	Collector/s and collector number	Date collected
EXT049‐01	JF0657	Cult Ngāti Kuri Nursery Te Paki ex. unknown	N/A	Genavee Rhodes	5/10/2023
EXT049‐02	BV0767	Cult Ngāti Kuri Nursery Te Paki ex. unknown	N/A	Genavee Rhodes	5/10/2023
EXT049‐03	20170833	Cult Auckland Botanic gardens ex. Unuwhao Sample ID 5	N/A	Ella Rawcliffe	12/10/2023
EXT049‐04	3	Unuwhao	CHR 597392	P J de Lange 12607 and A J Townsend	15/04/2015
EXT049‐05	5	Unuwhao	N/A	P J de Lange	15/04/2015
EXT049‐06	12	Kohuroa	N/A	P J de Lange	15/04/2015
EXT049‐07	16	Kohuroa	N/A	P J de Lange	15/04/2015
EXT049‐08	17	Tahae	N/A	P J de Lange	15/04/2015
EXT049‐09	270	Unuwhao	CHR 597394	P J de Lange 12605 and A J Townsend	12/04/2015
EXT049‐10	271	Unuwhao	CHR 597393	P J de Lange 12606 and A J Townsend	12/04/2015
EXT049‐11	281	Kohuroa	AK 357258	P J de Lange A Townsend	14/04/2015
EXT049‐12	286	Kohuroa	CHR 597387	P J de Lange 12637 and A J Townsend	14/04/2015

Abbreviations: Cult, cultivated; ex., location where cultivated samples were originally collected from or may be unknown.

**TABLE A2:** Raw ONT sequencing outputs for the two rātā Moehau libraries.

Sequencing data (ONT)	Batch 1	Batch 2
Mean read length (bp)	14,259.4	21,303.7
Median read length (bp)	12,543.0	17,485.0
Mean read quality	12.4	12.1
Median read quality	13.0	12.7
Total reads	1,568,743	1,005,856
Read length N50 (bp)	21,330	30,268
Total bases (Gb)	22.4	21.4

Abbreviations: Bp, base pairs; Gb, gigabases.

**TABLE A3:** Results of repeat annotation for the rātā Moehau genome assembly.

Repeat group	Repeat subgroup	Masked (bp)	Proportion of genome (%)
DNA	Academ‐1	53	< 0.0001
DNA	CMC‐Chapaev	44	< 0.0001
DNA	CMC‐EnSpm	99,959	0.0358
DNA	CMC‐Transib	252	0.0001
DNA	Crypton‐V	66	< 0.0001
DNA	Dada	78,379	0.0281
DNA	Ginger‐1	226	0.0001
DNA	IS3EU	1703	0.0006
DNA	Kolobok‐T2	1854	0.0007
DNA	MULE‐MuDR	4,274,140	1.5302
DNA	MULE‐NOF	308	0.0001
DNA	Maverick	1352	0.0005
DNA	Merlin	1100	0.0004
DNA	N/A	72,348	0.0259
DNA	P	212	0.0001
DNA	PIF‐Harbinger	583,322	0.2088
DNA	PIF‐ISL2EU	142	0.0001
DNA	PiggyBac	201	0.0001
DNA	TcMar	90	< 0.0001
DNA	TcMar‐ISRm11	480	0.0002
DNA	TcMar‐Mariner	563	0.0002
DNA	TcMar‐Pogo	48	< 0.0001
DNA	TcMar‐Tc1	84,940	0.0304
DNA	TcMar‐Tc2	38	< 0.0001
DNA	TcMar‐Tc4	131	< 0.0001
DNA	TcMar‐Tigger	457	0.0002
DNA	Zisupton	962	0.0003
DNA	hAT	1641	0.0006
DNA	hAT‐Ac	996,195	0.3567
DNA	hAT‐Blackjack	114	< 0.0001
DNA	hAT‐Charlie	3970	0.0014
DNA	hAT‐Tag1	2,356,862	0.8438
DNA	hAT‐Tip100	1226	0.0004
DNA	hAT‐hobo	553	0.0002
DNA	hAT N/A	319,281	0.1143
DNA	Total	8,883,212	3.1803
LINE	CR1	505	0.0002
LINE	CR1‐Zenon	126	< 0.0001
LINE	Dong‐R4	72	< 0.0001
LINE	I	8172	0.0029
LINE	I‐Jockey	339	0.0001
LINE	L1	4,437,483	1.5887
LINE	L1‐Tx1	500,050	0.1790
LINE	L2	2,872,792	1.0285
LINE	Penelope	913	0.0003
LINE	R1	53	< 0.0001
LINE	R1‐LOA	62	< 0.0001
LINE	R2	424	0.0002
LINE	R2‐NeSL	52	< 0.0001
LINE	RTE	1500	0.0005
LINE	RTE‐BovB	139,474	0.0499
LINE	RTE‐RTE	174	0.0001
LINE	RTE‐X	202	0.0001
LINE	Rex‐Babar	198	0.0001
LINE	Total	7,962,591	2.8507
LTR	Caulimovirus	16,154,790	5.7837
LTR	Copia	15,413,615	5.5183
LTR	DIRS	24,180	0.0087
LTR	ERV1	143,807	0.0515
LTR	ERVK	131,462	0.0471
LTR	ERVL	818	0.0003
LTR	ERVL‐MaLR	308	0.0001
LTR	Ty3	21,074,909	7.5451
LTR	N/A	607	0.0002
LTR	Ngaro	2123	0.0008
LTR	Pao	6574	0.0024
LTR	Total	52,953,193	18.9580
Low complexity	N/A	1,365,998	0.4890
Rolling Circle	Helitron	3,241,630	1.1606
Retroposon	L1‐dep	71	< 0.0001
SINE	5S‐Deu‐L2	68	< 0.0001
SINE	ID	167	0.0001
SINE	N/A	27	< 0.0001
SINE	Total	262	0.0001
Satellite	N/A	11,892	0.0043
Simple repeat	N/A	4,600,539	1.6471
Unknown	N/A	24,307,054	8.7023
Unspecified	N/A	41	0.0000
Unclassified	Total	24,307,095	8.7023
rRNA	N/A	110,419	0.0395
scRNA	N/A	105	< 0.0001
snRNA	N/A	22,216	0.0080
tRNA	N/A	20,003	0.0072
Small RNAs	Total	152,743	0.0547
Total	Total	103,479,226	37.0471

Abbreviations: LINE/SINE, long/short interspersed nuclear element; LTR, long tandem repeat; N/A, unspecified repeat subgroup; rRNA, ribosomal RNA; scRNA, small conditional RNA; snRNA, small nuclear RNA; tRNA, transfer RNA.
